# Stress Distribution Profile Imaging With Spectral Fabry-Perot Interferometry in Thin Layer Substrates for Surface Micromachining

**DOI:** 10.3390/mi8100294

**Published:** 2017-09-30

**Authors:** Adam Inzelberg, Yoav Linzon

**Affiliations:** School of Mechanical Engineering, Faculty of Engineering, Tel Aviv University, Tel Aviv 69978, Israel; adam@mail.tau.ac.il

**Keywords:** optomechanics, interference spectroscopy, thin films, local stress evaluation

## Abstract

We have used spectral two-layer interferometry (STLI) imaging for estimation of the stress distribution profiles (SDPs) in thin film substrates, enabling fast and reliable all-optical methodology for the evaluation of pre-stress topography profiles in silicon wafers deposited with thin films. Specifically, in polycrystalline silicon (PS) and silicon nitride (SN) thin films, we demonstrate a nondestructive, systematic, and robust capability for consistent stress distribution profile (SDP) evaluation relying on STLI. In particular, for PS and SN devices, the SDP estimation is consistent and is compared with complementary characterization of the films.

## 1. Introduction

Thin film devices consisting of materials such as polysilicon (PS) or silicon nitride (Si${}_{3}$N${}_{4}$, SN) are frequently used in micro-electronics, micro-photonics, and micro-machining industries and basic research incorporating these emerging fields. Typically, thin films can be grown using furnace-based methods such as physical vapor deposition (PVD) or low-pressure chemical vapor deposition (LPCVD). When a PVD/LPCVD process is not optimal, or when it is so desirable, non-uniform stress builds on the surface, mostly at the edges and center of the substrate. Significant residual stress may lead to defects and/or non-uniformities in the devices defined within the wafer. Many studies have elaborated on residual stress measurement in diverse configurations of silicon devices. In their recent review, Rossini et al. [1] discussed the different techniques to measure stress in thin films (e.g., PS or SN films). Typically, the distinction is between two categories of characterization methods: non-destructive versus destructive. These methods were summarized and compared by He [2]. General non-destructive methods include X-ray diffraction [3], neutron diffraction [4], and ultrasonic [5] measurements. In particular, as silicon is transparent in the near-infrared wavelengths (1100–1500 nm), methods reliant on photo-elastic polariscopy are popular in Si-based film stress evaluation. Danyluk and co-authors [6,7] have developed a far-field polariscope with a combination of phase-stepping and fringe multiplication to achieve record high stress distribution characterized over relatively large areas using infrared photoelasticity, where silicon wafers grown or deposited using various methods have been analyzed. There are some challenges in the popular photoelasticity-based characterization. Firstly, it consists of many components (including expensive polarization manipulators), and therefore is limited in size. Secondly, it uses invisible infrared wavelengths, requiring specialized sources and cameras. Lastly, the acquisition and processing runtime is long: typically 30–60 s [6,7]. In comparison with the previously mentioned methodologies for stress-profile evaluation, multi-wavelength analysis of spectrally resolved optical imaging is of particular interest, as it provides a non-destructive and potentially rapid film characterization. Here we demonstrate a new stand-alone spectrally resolved two-layer interferometry (STLI) imaging setup which is capable of extracting the full stress distribution profiles (SDPs) in thin film substrates and micro-devices defined within, achieving high accuracy and rapid processing. This method provides a rapid non-destructive setup for systematic SDP estimation in a multitude of substrates and films. Although there are many systems involving the STLI technique, the context of direct residual stress evaluation shown here has not been used before to the best of our knowledge. The implemented system is not fundamentally limited in size, may acquire five samples in a 30 s time-frame, has only five major optical components, and operates in the visible spectrum.

## 2. Experimental Setup and Theoretical Scheme

**Figure 1:**
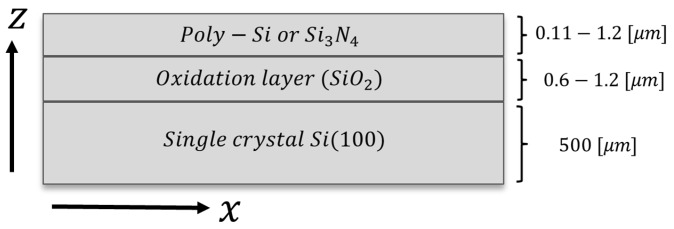
Cross-section of thin film layered structure. Stress distribution is assumed on the top layer.

The cross-section compositions and layer thicknesses in all tested wafers are illustrated in Figure 1. Each tested device has a 10.6 cm (4 in) diameter and is composed of three optically distinct layers. The substrate is a single crystal (400 μm thick) Si (100) substrate, a layer that can be considered much thicker than the middle and top layers as an important part of the analysis scheme. The middle layer is an oxidation layer (SiO${}_{2}$). The top layer is the active film inspected for stress profiling. Here we tested both poly-Si (PS) and silicon nitride (SN) films deposited using LPCVD at 800 ${}^{\circ }$C and 26 Pa atmosphere, where significant residual stresses persist in the top layer.

The STLI is based on multi-beam (Fabry–Perot) interference [8,9]. The system includes a collimated beam entering the cavity bordered by two reflective surfaces, Poly-Si at the upper surface, and highly-reflective Si (100) substrate at the bottom. While the typical interferometer system related to MEMS devices includes vacuum between these layers after oxidation layer release, here the dielectric material–SiO${}_{2}$–serves as the cavity medium. When this system is combined with a tunable band-pass filter scanning the input beam spectrum, STLI measurements are performed.

**Figure 2:**
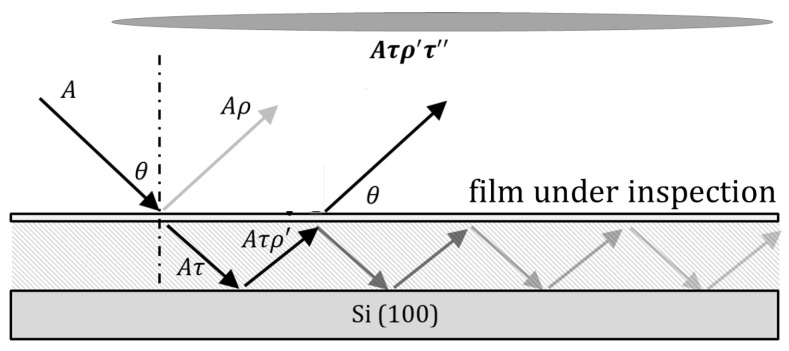
Two-layer interferometer and its application.

Figure 2 illustrates the interference geometry. In STLI, a monochromatic input beam (intensity *A*) traversing through the top and middle layer is partially transmitted (τ) and partially reflected (ρ) in each pass. Only an evanescent wave component persists through the infinitely thick and highly reflective substrate, and total internal reflection approximation is assumed for this interface. An input beam goes through multiple-beam interference via the cavity defined within the layered structure indicated in Figure 1. The entire emerging reflected radiation is focused via a wide-field imaging lens.

Following multiple-layer analysis, the relation between reflectance components and the refractive indexes of the composite layers reads [8]:

(1) $$\left(\begin{array}{c} a\\ b \end{array}\right)=M_{1}M_{2}\left(\begin{array}{c} 1\\ n_{s} \end{array}\right)$$

where each matrix $M_{i}$ represents the optical properties of the *i*th layer, a,b are scalars acquired from the reflectance equation $R=|\frac{a-b}{a+b}{|}^{2}$ and $n_{s}$ is the known substrate refractive index [8]. The complex coefficients *a* and *b* can always be unambiguously acquired from the second-order algebraic Equation (1) , where the subsequent scalar reflectance *R* includes the effects of both magnitudes and phases of the interfering parts within the interferometer [8]. We note in passing that under high deposition temperatures studied here, the film is usually assumed to include residual stress, whereas the oxidation layer stress is negligible [2]. This is further justified in the discussion section. Subsequent image processing to the reflectance maps produces refraction index maps as a function of wavelength. Then, by calculating the difference between a local refractive index and the theoretical value of unstrained reference refractive index, the stress is calculated via the photoelastic relation [9]:

(2) $$n_{i}\left(\lambda \right)=n_{0}\left(\lambda \right)-B_{1}S_{x}-B_{2}\left(S_{y}+S_{z}\right)$$

where $n_{0}\left(\lambda \right)$ is the unstressed film’s refractive index at wavelength λ, $n_{i}\left(\lambda \right)$ is the corresponding measured refractive index in the stressed film, $B_{j}$ are the stress-optical coefficients in direction *j*, and $S_{j}$ are the corresponding axis-dependent stresses. The stress-free state used in Equation (2) was calibrated in each pixel directly from reference image data. Reference refractive indexes and stress-optical coefficients were accounted for using the dispersion measurements by Green and Keevers for PS [10] and by Philipp for the case of SN [11]. An isotropic stress-optical value of $B_{1}=B_{2}=2.793\times 10^{-11}$Pa${}^{-1}$ was used for PS and $B_{1}=B_{2}=1.42\times 10^{-11}$Pa${}^{-1}$ for SN [9].

**Figure 3:**
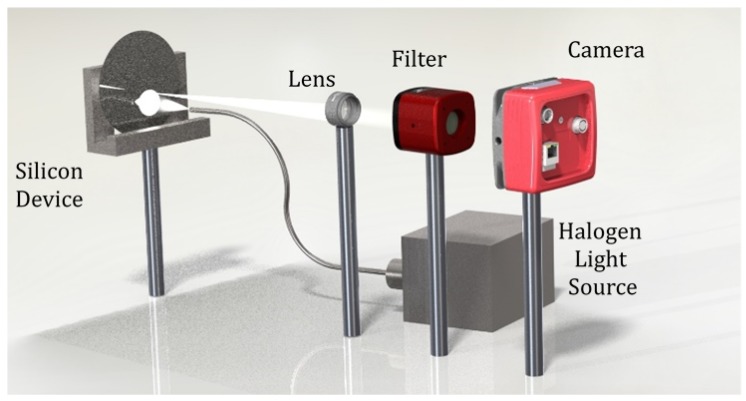
Schematic diagram of principal system components.

A schematic of the setup is shown in Figure 3. The STLI system consists of five principal components: Highly reflective silicon device (composition corresponding to Figure 2); Halogen (white light) 750 W illumination source; 75 mm wide-field converging lens; Liquid crystal tunable optical filter with a fixed 5 nm bandwidth in the visible spectrum (420–730 nm, Thorlabs KURIOS-WL1/M, Thorlabs, Inc., Newton, NJ, USA); and an 8 megapixel (MP), 10,248-level gray intensity scientific camera (Thorlabs 8050M-GE-TE, Thorlabs, Inc.). The filter selection is followed by a camera correction to adjust for altered focal plane position.

White light is emitted from the Halogen source toward the composite substrate at 45 degrees wide-field illumination. STLI light reflected from the surface (Figure 2) is collected through the lens and imaged on the camera plane following transmission through the filter aperture. In imaging condition, the filter is scanned over the visible range. Surface images are collected in conjuncture with filter scanning followed by focal point correction of the camera position in each wavelength. Dark environment is used during data collection in order to minimize background intensity levels and noise. Wavelength selection and camera operation and positioning are controlled through an autonomous program (LabView 2015, National Instruments, Austin, TX, USA) performing data acquisition followed by data analysis in real time. A MATLAB code operating on the gathered image-set performs the following analysis: after image matrix loading, the user indicates a reference position on the surface where stress-free areas of the film exists for the reference point. Intensity threshold normalization, noise reduction, and optical calculations reliant on Equations (1) and (2) are then performed for the rest of the surface. The total run-time including sample collection (16 wavelengths through 420–730 nm, with 20.5 nm intervals), data analysis, and output of refractive index and stress distribution profile was evaluated to be 2 min in total for the entire surface. The computation times over 8 MP coordinates may differ significantly working on different computers depending on available computing resources. However, the sample collection time cannot be shortened, and is limited by filter operation, camera positioning, and image acquisition rate. For a quantitative verification, a numerical simulation was performed in COMSOL multiphysics (v5.1, COMSOL Inc., Stockholm, Sweden), where the LPCVD process in the layered structure formation was simulated and compared to the experimental stress profiles.

## 3. Results and Discussion

**Figure 4:**
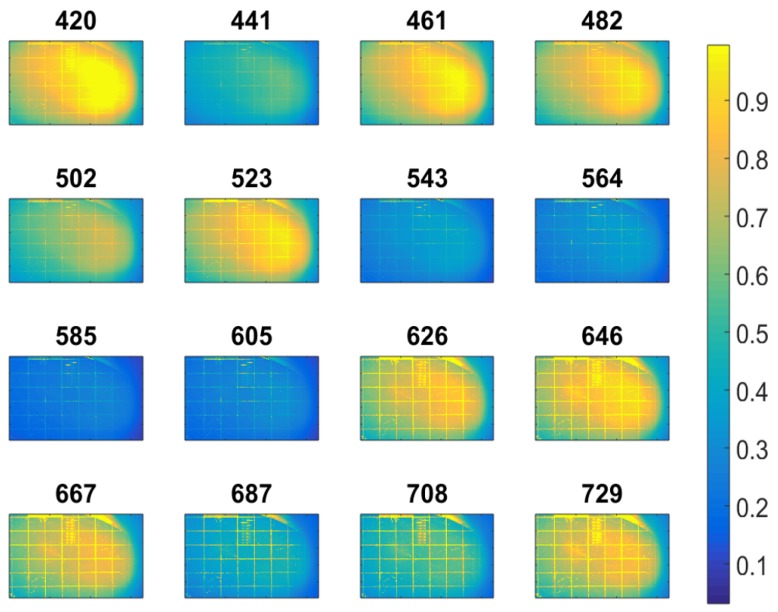
Surface reflection images at different wavelengths (16 filter settings) in the polysilicon (PS) device. Numbers indicate the central wavelength transmitted and the side colorbar denotes the relative intensity *R* normalized to 1.

We have quantitatively checked the operation of the setup over 8–10 different wafers of PS and SN, grown within different furnace temperatures, and consistent stress distribution profiles have been measured with comparable results that qualitatively scale in magnitude with the growth temperature. Here we will present the most significant cases of the stress profiles estimated at the highest growth temperatures of 600 ${}^{\circ }$C for PS film and 592 ${}^{\circ }$C for the SN film. Figure 4 shows the measured surface images in PS, at 16 wavelengths. Samples have been taken in subsequent sets ranging from 420 nm to 729 nm with 20.5 nm intervals. Differences arising from features related to STLI can be observed in the raw data even before detailed analysis.

**Figure 5:**
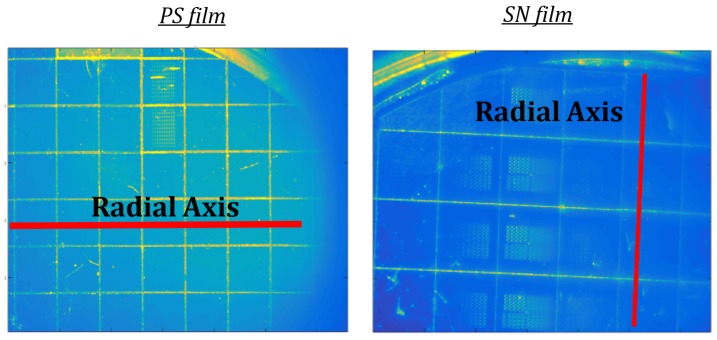
Polysilicon (PS, left) and silicon nitride (SN, right) device surface images and radial axes chosen for the stress distribution profile (SDP) cross-sections evaluated.

Figure 5 displays two images corresponding to PS (left) and SN (right) surfaces, in the wafers chosen, where red lines indicate the radial axes chosen for stress assessment in the following. Color represents intensity levels.

**Figure 6:**
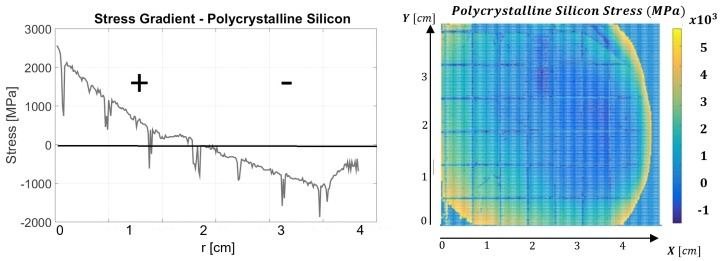
SDP evaluated along a radial line for the PS device (left) and the full surface stress profile (right) .

In both PS and SN devices, the SDPs were evaluated several times, as derived from the 16 refractive index profiles (RIPs). Figure 6 shows the full extracted surface SDP (right) and the stress gradient across the chosen radial line in the PS device. Low spikes in the profile occured due to diced squares in the wafer, and their existence does do not inflict overestimation in the extraction algorithm. We note that the wafers were diced to simulate realistic operation of the system as a wafer inspection tool for MEMS applications before and after photolithography. The wavelength 585 nm has been dominant in stress evaluation in both PS and SN devices. This dominant wavelength was due to the best transmission exhibited by the electro-optical filter (see Filter component in Figure 3), and we anticipate that a tunable filter with uniform transmission throughout the whole visible spectrum without resonances—which is not yet available commercially—would not yield dominant wavelengths. As observed in Figure 6, compressive stresses ranging between 1000 and 1500 MPa are estimated near the wafer edges (large r>2 cm), decreasing to zero stress and becoming an increasing tensile stress toward the wafer center peaking at 2500 MPa. The profile exhibits tensile stress near the center and compressive stress close to the wafer edges.

**Figure 7:**
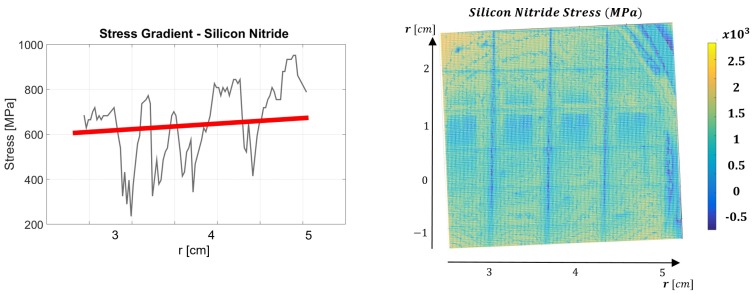
SDP along a radial line for the SN device (left) and estimated surface stress profile (right). Red line corresponds to stress value obtained from independent wafer-bow measurement.

In Figure 7 are shown the SDP profiles corresponding to the SN device, where the left panel again corresponds to the chosen radial cross-section, whereas in the right panel is shown the full stress map. Low values at 3.3, 3.9, and 4.4 cm again appear to the visible dicing of the wafer. In this case as well, dicing has been introduced to simulate realistic inspection conditions. The extracted profiles show a consistent stress profile along the radial line, where the tensile stress ranges between 220 and 900 MPa. The measured values agree with previous characterization of this wafer independently, evaluated using wafer-bow technique [12], and yielding a tensile stress value of 600–650 MPa in this wafer (red line in Figure 7). Wafer-bow data, obtained successfully in the SN case, nonetheless yielded an irreversible fractural damage in the PS case prior to data acquisition when attempted. It is possible that these results can be attributed to the fragility of the stress profile which showed high variation in sign and magnitude in the latter, while in the former the stress was tensile throughout with a slow gradient.

In direct comparison between the two methods of stress estimation (Figure 7, left), we emphasize that the wafer-bow technique gives an estimation of 600–650 MPa throughout the profile, averaged more toward the wafer center than the edges with less localized sensitivity [12], whereas our optical characterization is potentially more sensitive to local variations and changes in the range 400–900 MPa. The quantitative relative agreement between the two methods around the wafer center ( *r* = 2.5 cm) and discrepancy near the edges is thus plausible after removal of the stress deeps in the optical measurement, attributed to the diced regions. In both cases, the stress is always positive corresponding to the tensile case.

In order to obtain an indication relating to the accuracy of our estimations, we compared the experimental results to a COMSOL multiphysics (v5.1) simulation of the LPCVD growth process at a temperature of 670 ${}^{\circ }$C, using the solid mechanics coupled with heat transfer in solids modules through multiphysics boundary conditions. Table 1 highlights the physical parameters taken for each of the three materials, of the geometrical attributes corresponding to Figure 1.

While the SFPI can be generically sensitive to either of the layers and to bi-morph effects in combined stresses, Table 1 shows that in the specific cases studied, both thermal conductivity and thermal expansion coefficients of the intermediate oxidation layer are 1–2 orders of magnitude smaller than the film layer. In the case of PS film, one can therefore assume that most of the stress characterized is related to the film layer alone. In more general cases, however, more special care should be attributed to the interpretation of the SDP result, with a possibly more elaborate model than the one assumed in Equation (2).

**Figure 8:**
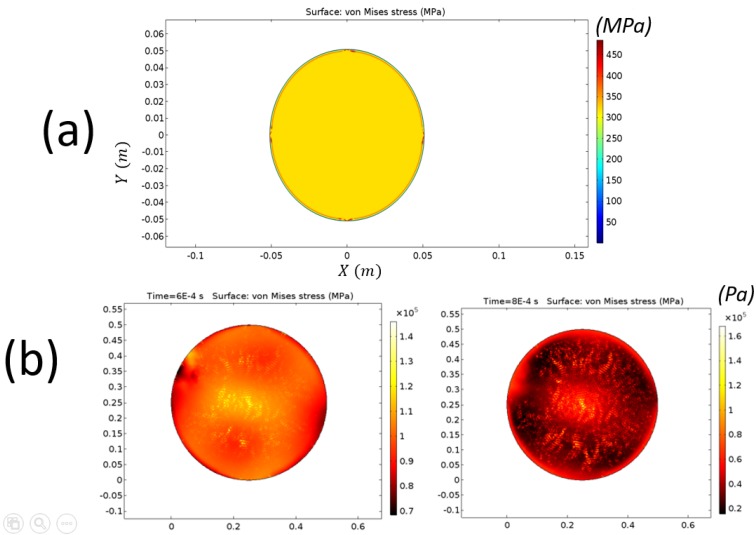
SDPs for the entire 10 cm (4 in) diameter wafer surface as obtained from COMSOL numerical simulation along a radial line for the SN device. (**a**) stationary solver; (**b**) time-dependent solution, after thermalization time of 60 ms (left) and 80 ms (right).

Two different solutions were highlighted. Numerical solutions are shown in Figure 8. The stationary solution for PS (Figure 8a) shows a stress profile with relatively high compression values at the edges (450–600 MPa), which was decreasing along the profile, and −300 MPa at the edges. On the other hand, a time-dependent solution (Figure 8b) exhibits a very short time frame with high stresses appearing throughout the surface during initial baking, exhibiting underestimated values for the stress distribution as compared to the experimental and steady-state estimations. In the latter case, the distribution of stress along the profile correlated with the transition from tension in the wafer center to compression in the edges, consistent with the experimental observations. In direct comparison with the experimentally deduced stress-profiles, the former stationary solution (Figure 8a) reproduced the correct order of magnitude, whereas the latter time-dependent solution (Figure 8b) reproduced the correct stress gradient being tensile near the wafer center and compressive near the wafer edges.

## 4. Conclusions

Using spectral two-layer interferometry imaging, an all-optical non-destructive stress distribution profile measurement method has been developed and demonstrated in silicon wafers deposited with either polycystalline Silicon or silicon nitride films with high residual stresses on sacrificial oxide layers. The technique presented relies on Fabry–Perot interferometry, and features full non-destructive automatic operation, with short run times for both data acquisition and analysis. It can be especially useful for fast stress evaluation in surface micromachining applications, in which devices defined lithographically and released can depend critically on the magnitude and sign of the film pre-stress. The results show plausible SDPs in both device compositions. To summarize, the technique presented enables fast quantification and quality assurance in thin-film characterization. 

## Figures and Tables

**Table 1:** Physical properties in layers used in the numerical model.

Parameter	Substrate-Si (100)	Oxidation Layer-SiO${}_{2}$	Film Layer-PS
Density (kg/m${}^{2}$)	2330	2203	2330
Young’s modulus (GPa)	110	78	169
Poisson’s ratio	0.19	0.17	0.22
Thermal expansion coefficient (1/κ)	2.5 × 10${}^{-6}$	0.35 × 10${}^{-6}$	2.9 × 10${}^{-6}$
Thermal Conductivity (W/(m × K))	148	1.4	34
Heat Capacity (J/(kg × K))	712	700	678
